# Successes and failures of angiogenesis blockade in gastric and gastro-esophageal junction adenocarcinoma

**DOI:** 10.3389/fonc.2022.993573

**Published:** 2022-09-23

**Authors:** Massimiliano Salati, Francesco Caputo, Alessandro Bocconi, Sara Cerri, Cinzia Baldessari, Federico Piacentini, Massimo Dominici, Fabio Gelsomino

**Affiliations:** ^1^ Division of Oncology, Department of Oncology and Hematology, University Hospital of Modena, Modena, Italy; ^2^ PhD Program Clinical and Experimental Medicine, University of Modena and Reggio Emilia, Modena, Italy

**Keywords:** gastric cancer, angiogenesis, targeted therapy, precision medicine, clinical trials, gastro-esophageal adenocarcinoma

## Abstract

Gastric and gastro-esophageal junction adenocarcinoma (GEA) remains a considerable major public health problem worldwide, being the fifth most common cancer with a fatality-to-case ratio that stands still at 70%. Angiogenesis, which is a well-established cancer hallmark, exerts a fundamental role in cancer initiation and progression and its targeting has been actively pursued as a promising therapeutic strategy in GEA. A wealth of clinical trials has been conducted, investigating anti-angiogenic agents including VEGF-directed monoclonal antibodies, small molecules tyrosine kinase inhibitors and VEGF-Trap agents both in the resectable and advanced setting, reporting controversial results. While phase III randomized trials testing the anti-VEGFR-2 antibody Ramucirumab and the selective VEGFR-2 tyrosine kinase inhibitor Apatinib demonstrated a significant survival benefit in later lines, the shift of angiogenesis inhibitors in the perioperative and first-line setting failed to improve patients’ outcome in GEAs. The molecular landscape of disease, together with novel combinatorial strategies and biomarker-selected approaches are under investigation as key elements to the success of angiogenesis blockade in GEA. In this article, we critically review the existing literature on the biological rationale and clinical development of antiangiogenic agents in GEA, discussing major achievements, limitations and future developments, aiming at fully realizing the potential of this therapeutic approach.

## Introduction

Gastric and gastroesophageal junction adenocarcinoma (GEA) remains a considerable health problem, ranking fourth as the leading cause of cancer-related death globally, with more than 700,000 deaths in 2020 (1). Therapeutic advances occurred over the recent years have led to incremental improvements in patient outcomes, and the advent of next-generation sequencing technologies have improved our biological understanding of GEA, unravelling profound molecular heterogeneity and potential vulnerabilities (2). For patients with resectable GEA, taxane-based triplet has outperformed anthracycline-based therapy as the reference regimen in the perioperative setting, leading to a 10%-increase in the curability rate (3). In the advanced disease setting, the growing number of novel effective drugs, including cytotoxics, targeted agents and checkpoint inhibitors and the improved patients and molecular selection are making the continuum of care a reality in GEA, with some patients experiencing an unexpected survival (4–10).

Among the most successfully exploited targets in GEA is angiogenesis with the VEGFR2-directed antibody ramucirumab being the second targeted agent to be approved, either alone or combined with paclitaxel, for advanced GEA failing prior platinum and fluoropyrimidine chemotherapy (11). Angiogenesis is a well-established cancer hallmark and angiogenesis-related factors, among which the VEGF/VEGFR axis is the most extensively studied, therapeutically validated and characterized, are overexpressed in many cancers, including GEA, and correlate with a poor prognosis (12–14). This has soon made angiogenesis an attractive target for therapeutic interventions and antiangiogenic therapy a component of standard treatment across a variety of cancer types. However, aside from the established role for ramucirumab in the second-line setting, the targeting of angiogenesis has provided controversial results in GEA. While early phase trials displayed promising antitumor activity, the near totality of them failed to prolong survival in randomized-controlled phase III trials in both resectable and advanced disease (15). Moreover, the lack of reliable predictive biomarkers has further hindered the optimization of angiogenesis blockade in GEA. Here, we extensively reviewed the biological rationale, the successes and failures from clinical trials as well as future developments for angiogenesis blockade in GEA.

## The biological rationale for angiogenesis targeting in GEA

Angiogenesis enables the generation of a neovasculature from pre-existing blood vessels sustaining tumor progression and invasion since the early stages of cancer development (13). During this multistage process of invasive cancers indeed, malignant cells acquire the capability of activating an “angiogenic switch” through the unbalance in favor of pro-angiogenic factors. Compelling evidence showed that this “angiogenic switch” is regulated by stimulatory and inhibitory factors, which act through signaling proteins and transmembrane receptors on the surface of vascular endothelial cells. The most extensively characterized angiogenic regulator is the vascular endothelial growth factor (VEGF) together with its receptors (VEGFR) (14). The VEGF family consists of six protein isoforms called VEGF-A, -B, -C, -D, -E, -F, and placental growth factors -1 and -2 (PIGF). VEGF binds to transmembrane tyrosine kinase receptors VEGFR in three members VEGFR-1 and VEGFR-2 that present high affinity for VEGF-A, and VEGFR-3, specific for VEGF-C e VEGF-D (16). Upon VEGF binding to the extracellular domain of VEGFR, the receptor undergoes a dimerization process, phosphorylation of the tyrosine kinase domain, and subsequent activation of an intracellular pathway that in turn results in changes in cell morphology, cytoskeleton alteration, and stimulation of endothelial cell survival, proliferation and migration (13). While the role of VEGFR-1 in the angiogenesis process remains unclear, with some studies suggesting it as a negative regulator of VEGF activity and others showing an active role in the recruitment of endothelial cell progenitors, the VEGFR-2 represent the main actor in this process (14). Indeed, thanks to the binding with the ligand VEGF-A, VEGFR-2 mediates proliferation, invasion, migration and survival of endothelial cells via the MAP-kinase signalling pathway (17, 18). Instead, the VEGFR-3, which is expressed only in lymphatic endothelial cells in adults, preferentially binds VEGF-C and VEGF-D and its activation and up-regulation enhance tumor lymphangiogenesis and lymph node metastasis (19). In addition to causing the sprouting of new vessels, the VEGF/VEGFR axis induces increased vascular permeability, which results in the leakage of plasma protein, fibrin deposition and ultimately in maintaining the proangiogenic make-up of tumor microenvironment (13, 14). Notably, the aberrant neovasculature resulting from cancer angiogenesis is also responsible for the malignant ascites typically shown in advanced-stage GEA (20, 21). These effects are also dependent on the influence of the VEGF/VEGFR signalling system on the proteins expressed on tight and adherens junctions, as VE-cadherin, occludin, and claudin 5. During angiogenesis, after interaction between VEGF and its receptor, a process including the phosphorylation of VE-Cadherin and its internalization into clathrin-coated pits leads to the disruption of the architecture of endothelial junctions, allowing for the passage of molecules and cells (22). Similarly, VEGF induces phosphorylation of tight junction proteins occludin and Zonula Occludens 1, increasing vasculary permeability, as shown both in pathological brain and ocular conditions, as well as in cancers (23, 24).

In recent years, lights have been shed on the molecular mechanisms underpinning VEGF/VEGFR-mediated angiogenesis, among which the hypoxia-inducible factor-1 (HIF-1) activation through the hypoxic neoplastic milieu is the best-established one. Following its translocation to the cellular nucleus, HIF-1 binds the VEGF promoter and leads to an increase VEGF transcription (25, 26). Additionally, the EGFR/HER2 system has been shown to induce neuropilin-1 and VEGFR expression in several cancer types, including GEA (27). On the other hand, it has been shown that Notch is a negative regulator of the VEGF/VEGFR axis (28, 29) activity as an increased in VEGFR-2 and VEGFR-3 expression is seen in stalk cells upon abrogation of Notch signalling. Wnt5a may suppress angiogenesis via non-canonical signaling by reducing both EC proliferation and capillary length (30).

Another signaling pathway implicated in angiogenesis is represented by the angiopoietin/tie cascade, which is made of four different angiopoietins (Ang-1,-2,-3,-4), which bind the tyrosine kinase receptor Tie-2. Ang-2, which is involved in vessel maturation, migration, adhesion and survival of endothelial cells, has a crucial role in tumor angiogenesis (31). Again, the FGF (fibroblast growth factor)/FGFR pathway, which controls tumor angiogenesis through the activation of the AKT-pathway, deserves to be mentioned (32). An additional player described in GEA is the non-classical activator tryptase that activates proteinase-activated receptor-2 (PAR-2) and stimulates VEGF production (32, 33). The role of this tryptase and the potential usefulness of anti-angiogenic therapy in gastric cancer is demonstrated by the evidence that infiltrating mast cells, that released tryptase, was related to microvascular density (34).

Taken together, these findings have demonstrated that GEA displays a high angiogenic potential enabled mostly by the VEGF/VEGFR axis and have highlighted a robust and consistent biological rationale for the targeting of angiogenesis in GEA. One of the first proof-of-concept experiences on the targeting of VEGF-driven angiogenesis was reported by Yuan et al. showing time-dependent vascular endothelial regression and changes in vessels permeability in human tumor xenograft treated with neutralizing anti-VEGF antibodies (35). Consistent with that, the anti-VEGF bevacizumab showed to reduce cell growth and tumor size in both in vitro and in vivo experimental models of GEA (36, 37). Analogously, the blockade of VEGFR-2 by means of a targeted monoclonal antibody has resulted in inhibited proliferation and increased apoptosis in preclinical models of GEA (38, 39). These effects were even more pronounced when the anti-VEGF agent was combined with HER2 inhibitors in mice (40). More interestingly, an attractive strategy was suggested by the inhibition of VEGFR-1 or VEGFR-2, which has been reported to enhance paclitaxel sensitivity by decreasing the chemoresistance-conferring elements HIF-1α and TUBB3 (41). Based on this promising preclinical evidence, the targeting of GEA-induced angiogenesis has rapidly advanced to clinical development with controversial study results, which are reviewed in the next chapter.

## Clinical development of antiangiogenic agents in GEA

**Figure 1:**
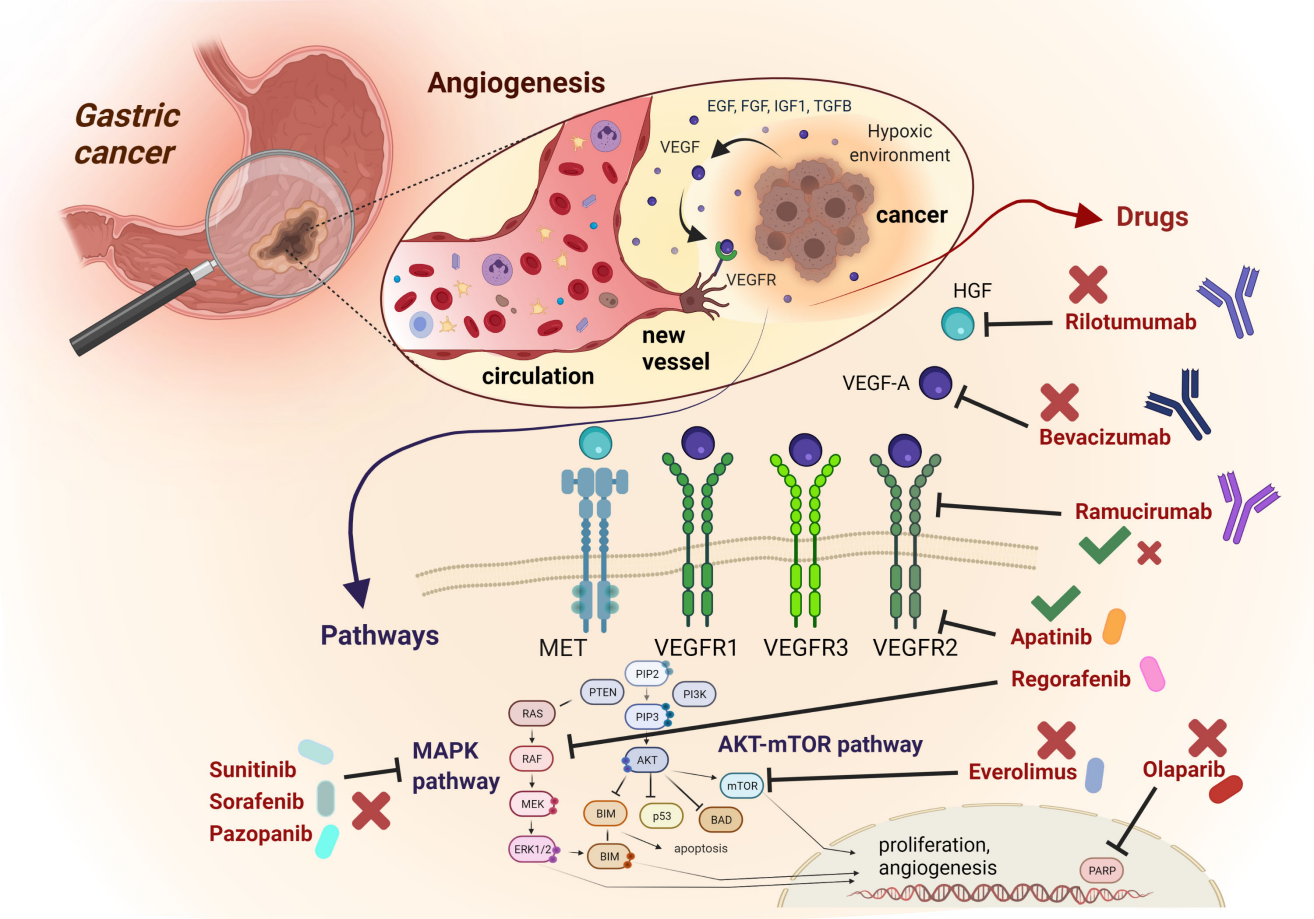
Schematic representation of most relevant angiogenic signalling pathways and related targeted compounds in GEA. The figure describes an enlargement of the gastric wall, with the most relevant mechanisms involved in angiogenesis, The anti-angiogenic drugs mentioned in the text are reported, with their targets and their related positive (green thick) or negative (red cross) results in clinical trials concerning GEA patients.

The crucial role played by angiogenesis in cancer progression and invasion, together with the promising results achieved through its pharmacological inhibition in preclinical models, have rapidly advanced the development of this therapeutic strategy to the clinical stage across a wide array of malignancies, including GEA. Although from one hand, the blockade of angiogenesis was the second success of precision medicine in GEA following HER2 inhibition, on the other hand the clinical development process of anti-angiogenic agents is studded with disappointing results. In this section, we discuss major positive and negative trials that have attempted angiogenesis blockade in GEA (**Figure 1**).

### Positive trials

Ramucirumab is a fully human IgG1 anti-VEGFR-2 monoclonal antibody, that exerts its action by preventing the binding of VEGF (A, C, D) to the receptor and blocking signaling activation in endothelial cells. Two pivotal trials demonstrated overall survival (OS) prolongation, leading to the approval of the first target agent in the setting of pre-treated GEA (6, 7). The REGARD study is a phase III randomized trial, in which advanced GEA patients whose disease progressed after first-line platinum/fluoropyrimidine-containing chemotherapy were randomly allocated (2:1) to either ramucirumab 8 mg/kg intravenously once every 2 weeks plus best supportive care (BSC) or placebo. The median OS was 5.2 months in the ramucirumab group and 3.8 months in the placebo group (hazard ratio [HR]=0.77, 95% CI 0.60-0.99; p=0.047) (6). Regarding treatment-related adverse events (AEs), the occurrence of hypertension was higher in the investigational arm than in the control arm (16% vs 8%), whereas rates of other AEs were similar between treatment groups (94% vs 88%). In the phase III RAINBOW trial patients with advanced GEA progressing on or within 4 months after upfront chemotherapy (platinum/fluoropyrimidine with or without anthracycline) were randomized 1:1 to receive ramucirumab 8 mg/kg intravenously once every 2 weeks or placebo, plus paclitaxel 80 mg/mq intravenously on days 1, 8, and 15 of a four-week cycle (7). The OS was significantly prolonged in the experimental arm compared to the control arm (median OS 9.6 months vs 7.4 months, HR=0.80, 95%CI 0.67-0.96, p=0.017). The most common grade 3 or higher AEs (occurring in more than 5% of patients) in the ramucirumab plus paclitaxel arm versus the paclitaxel arm were neutropenia, leucopenia (17% vs 7%), hypertension (14% vs 2%), fatigue (12% vs 5%), anemia (9% vs 10%), and abdominal pain (6% vs 3%). Given the results of these two phase III trials, ramucirumab has been approved by regulatory agencies either in monotherapy or in combination with paclitaxel for the second-line treatment of GEA.

Apatinib is an oral small molecule tyrosine kinase inhibitor that selectively targets VEGFR-2. In a phase III randomized-controlled trial, Chinese patients with advanced GEA failing two or more prior lines of chemotherapy, were assigned to apatinib 850 mg once daily or placebo (42). The median OS was significantly improved in the apatinib arm compared with the placebo arm (6.5 months vs 4.7 months, HR=0.709, 95%CI, 0.53-0.93, p=0.0156). The most frequently reported grade 3 to 4 non-hematologic AEs included hand-foot syndrome, proteinuria and hypertension. Based on these results, in 2014 Apatinib was approved in China in this setting. However, the subsequent phase III ANGEL trial failed to confirm the efficacy of apatinib in a global population (43) (**Table 1**).

**Table 1:** Major clinical trials reporting positive results for angiogenesis blockade in GEA.

Trial(Authors)	Phase	Line	Target	Treatment arms	Overall Survival^1^, months	Safety profile^2^
REGARD (Fuchs et al.)	III	II	VEGFR-2	Ramucirumab (n=238) vs Placebo (n=117)	5,2 vs 3,8 HR=0,776 (0,603-0,998) p=0,042	Hypertension (16% vs 8%)
RAINBOW (Wilke et al.)	III	II	VEGFR-2	Ramucirumab + Paclitaxel (n=330) vs Paclitaxel + Placebo (n=330)	9,6 vs 7,4 HR=0,807 (0,678-0,962) p=0,017	Neutropenia(41% vs 19%); hypertension (14% vs 2%); fatigue (12% vs 5%)
Li et al.	III	≥ III	VEGFR-2	Apatinib (n=176) vs Placebo (n=91)	6,5 vs 4,7 HR=0,709 (0,537-0,937) P=0,0156	HFS (8.5% vs 0%); Proteinuria (2.3% vs 0%); Hypertension (4.5% vs 0%)

^1^Investigational arm vs control arm.

^2^Grade 3-4 adverse events.

### Negative trials

The anti-VEGF-A humanized IgG1 monoclonal antibody bevacizumab, which is an evidence-based option in numerous cancer types including, among others, metastatic colorectal cancer, has been the most extensively investigated anti-angiogenic drug in GEA. Early phase II trials showed an encouraging activity for bevacizumab in combination with cytotoxic agents such as fluoropyrimidines, platinum derivatives, irinotecan and taxanes in metastatic GEA, with an overall response rate (ORR) in the range of 42-74%, a median progression-free survival (PFS) of 6-12 months and a median OS of 10.8-17.9 months (44–48). Despite this encouraging activity, the phase III advancement of bevacizumab to the first-line setting proved unsuccessful results in both the western and Asian patient population. In fact, in the Avastin in Gastric Cancer trial (AVAGAST), the addition of bevacizumab to standard first-line combination capecitabine-cisplatin improved PFS and ORR, yet this did not translate into an OS advantage (primary endpoint) (12.1 vs 10.2 months, HR=0.87; p=0.1002) in 774 patients from around the world. Among factors advocated to explain the failure of this large-scale trial is the heterogeneity of the enrolled population, as supported by the differential treatment efficacy recorded in Pan-American as opposed to Asian patients (HR for OS 0.63 vs 0.97, respectively) (49). Accordingly, the AVATAR trial conducted with the same study design in 202 Chinese patients, demonstrated a superimposable OS between the experimental and the control group (HR=1.11; p= 0.5567) (50). Although a biomarker analysis of the AVAGAST trial suggested a positive predictive value for high plasma VEFG-A and neuropilin-1 levels in the non-Asian subgroup, these preliminary findings have been neither validated nor developed further (51). Likewise, bevacizumab failed to prove its efficacy in the setting of resectable GEA. In fact, in the ST03 phase II-III trial, more than 1000 resectable GEA patients were randomly allocated to standard perioperative chemotherapy consisting of epirubicin, cisplatin and capecitabine plus or minus bevacizumab. The 3-year OS was 50.3% in the chemotherapy only arm and 48.1% in the chemotherapy plus bevacizumab arm (HR=1.08, 95%CI 0.91-1.29; p=0.36), with an increased risk of impaired wound healing in the bevacizumab arm (52).

Furthermore, despite ramucirumab having demonstrated its clinical activity in monotherapy or combined with paclitaxel in the second-line setting, the results of 3 randomized trials, including 2 phase II and one phase III, in the front-line treatment of GEA were surprisingly disappointing (53–55). In particular, RAINFALL study was an international, phase 3 trial in which patients with metastatic, HER2-negative GEA were randomized 1:1 to cisplatin plus capecitabine (or 5-FU) and either ramucirumab or placebo. The investigator-assessed PFS (primary endpoint) was significantly extended in the experimental arm as compared to the control arm (HR=0.75, 95%CI 0.60-0.93, p=0.0106; median PFS 5.7 vs 5.4 months). However, a sensitivity analysis based on a central radiological review did not confirm the investigator-assessed difference in PFS nor showed any difference in terms of OS between the two groups (55). This study raised some major concerns. Firstly, as previously reported, PFS does not appear to be a good surrogate endpoint for OS in advanced GEA (56). Secondly, the high rate of post-progression treatments (46% and 50%, in the ramucirumab and in the placebo arm, respectively) might have diluted the difference between the two groups in terms of OS. Thirdly, since ramucirumab has proved its efficacy when paired with a taxane for second-line treatment, it is unknown whether the addition of a taxane instead of the combination of cisplatin and a fluoropyrimidine in the first-line setting could have improved outcomes.

Ziv-aflibercept is a recombinant fusion protein that functions as a decoy receptor to bind VEGF-A, VEGF-B, and PIGF with a high affinity (57), which has been approved in combination with the FOLFIRI regimen in patients with colorectal cancer who were pre-treated with an oxaliplatin-contaning regimen. In a phase II randomized trial in patients with treatment-naive metastatic GEA, the addition of ziv-aflibercept to mFOLFOX6 did not demonstrate an advantage in terms of 6-month PFS (primary endpoint), RR and OS (58, 59).

Sunitinib is a multikinase inhibitor targeting multiple RTKs, including VEGFR1-3, PDGFR, KIT, RET, colony-stimulating factor 1 and FLT3 (60), which are involved in different malignancies, that showed poor efficacy in terms of ORR (less than 5%) in two phase II trials (61, 62) in pretreated patients with advanced GEA. Moreover, other studies testing the addition of sunitinib to chemotherapy in different treatment lines did not demonstrate any advantage in terms of PFS and OS (63, 64).

Similarly, other multikinase inhibitors, such as Sorafenib and Pazopanib, showed modest activity in the treatment of advanced GEA (65–69) (**Table 2**).

**Table 2:** Major clinical trials reporting negative results for angiogenesis blockade in GEA.

Trial(Authors)	Phase	Line	Target	Treatment arms	Overall survival^1^, months	Safety profile^2^
AVAGAST (Ohtsu A. et al.)	III	I	VEGF	Bevacizumab + chemo (n=387) vs chemo + placebo (n=387)	12,1 vs 10.1 HR=0,87 (0.73-1,03) p=0,1002	Neutropenia (35 vs 37%); anemia (10 vs 14%)
RAINFALL (Fuchs C et al.)	III	I	VEGFR-2	Ramucirumab + chemo (n=326) vs chemo + placebo (n=319)	11,2 vs 10,7 HR=0,96 (0,80-1,15) p=0,74	Neutropenia (26% vs 27%); Hypertension (10% vs 2%)
RILOMET-1 (Catenacci D. et al.)	III	I	HGF	Rilotumumab + chemo (n=304) vs chemo + placebo (n=305)	8,8 vs 10,7 HR=1,34 (1,10-1,63) p=0,003	Neutropenia (29% vs 32%); anemia (12 vs 14%); fatigue (10% vs 12%)
GOLD (Bang YJ et al.)	III	II	PARP	Olaparib + paclitaxel (n=263) vs paclitaxel + placebo (n=262)	8.8 vs 6,9 HR=0,79 (0,63-1,00) p=0,026	Neutropenia (30% vs 23%)
GRANITE 1 (Ohtsu A. et al.)	III	III	mTOR	Everolimus (n=439) vs Placebo (n=217)	5.4 vs 4.3 HR=0,90 (0,75-1,08) p= 0,124	Anemia (16% vs 13%) Decresead appetite (11% vs 6%); Fatigue (8% vs 5%)

^1^Investigational arm vs control arm.

^2^Grade 3-4 adverse events.

Rilotumumab is a fully human monoclonal antibody selectively targeting the hepatocyte growth factor (HGF). This pathway plays an important role in angiogenesis and tumor growth, with a synergistic action by HGF and VEGF on endothelial cells and also an increasing expression of angiogenic factors mediated by HGF (70, 71). For these reasons, inhibitors of HGF/c-Met signaling pathway are being developed to inhibit angiogenesis. Rilotumumab was investigated in phase III RILOMET-1 trial, in which more than 600 patients with untreated unresectable locally advanced or metastatic MET-positive GEA (defined as ≥25% of cancer cells with a membrane staining intensity of ≥1+) were randomly allocated to receive rilotumumab or placebo combined with antracycline-based chemotherapy (i.e. epirubicin, cisplatin and capecitabine). The investigational treatment was stopped prematurely after a reporting by the independent data monitoring committee of an increased number of deaths in the rilotumumab arm. The median OS was 8.8 months in the rilotumumab group compared with 10.7 months in the placebo group (stratified HR=1.34, 95%CI 1.10-1.63; p=0.003) (72).

Everolimus is an oral mTOR inhibitor, which demonstrated encouraging antitumor activity in a phase II study of previously-treated advanced GEA (73). mTOR is part of the phosphatidylinositol 3-kinase/Akt/mTOR signaling pathway, which is implicated in tumor angiogenesis (74). This pathway is involved in the regulation of HIF-1α, VEGF, as well as in the endothelial cells function, thus regulating tumor vascularization (75). In the phase III GRANITE-1 trial patients progressing after one or two chemotherapy lines were randomly assigned 2:1 to everolimus 10 mg/die or placebo, both given in combination with BSC. In this study, everolimus did not significantly prolong OS as compared to placebo, with a median OS of 5.4 months in the experimental arm and 4.3 months in the placebo arm (HR=0.90; 95%CI, 0.75-1.08; p=0.124) (76).

Interestingly, the PARP inhibitor olaparib has shown a significant improvement in OS when added to paclitaxel as second-line therapy in a phase II study enrolling Asian patients with advanced GEA, particularly in the molecular subset with ataxia-telangiectasia mutated protein (ATM)-negative tumors (77). In fact, PARP inhibitors have shown to have anti-angiogenic effects by inhibiting VEGF action (78).

Therefore, in the phase III GOLD study Asian patients with advanced GEA progressing after or during first-line chemotherapy were randomized 1:1 to oral olaparib plus paclitaxel or placebo plus paclitaxel. The OS did not differ between treatment groups neither in the overall patient population (median OS 8.8 months vs 6.9 months in the olaparib and placebo group, respectively, HR=0.79, 95%CI 0.63-1.00, p=0.026) nor in the ATM-negative population (12.0 months vs 10.0 months, HR=0.73, p=0.25) (77, 79).

### Ongoing studies

Regorafenib is an oral multikinase inhibitor that blocks several receptor tyrosine kinases implicated in angiogenic, oncogenic and stromal signalling. On the basis of the very promising results of the randomized phase II INTEGRATE trial, in which regorafenib proved to be effective in prolonging PFS in advanced, refractory GEA (80), an international phase III trial (INTEGRATE II, NCT02773524) is currently enrolling patients. Furthermore, two phase I/II studies are testing the combination of regorafenib with immune checkpoint inhibitors, including avelumab (REGOMUNE, NCT03475953) and nivolumab (REGONIVO, NCT03406871). Various ongoing clinical trials are currently evaluating the potential of ramucirumab in other settings or in combination with other drugs. RAMSES trial (NCT02661971) is a randomized phase III trial of perioperative FLOT plus or minus ramucirumab in resectable GEA, while RAMIRIS (NCT03081143) is comparing FOLFIRI plus ramucirumab versus paclitaxel plus ramucirumab. The ARMANI trial (NCT02934464) is a phase III trial comparing the first-line treatment continuation versus a switch maintenance strategy with paclitaxel plus ramucirumab. Fruquintinib is a selective oral inhibitor of VEGFR-1-3, which is currently under investigation in numerous clinical trials. FRUTIGA (NCT03223376) is a phase III study comparing paclitaxel plus Fruquintinib versus paclitaxel alone in the second-line setting.

**Table 3** summarizes the most promising ongoing clinical trials with anti-angiogenic in the treatment of GEA.

**Table 3:** Selected ongoing trials investigating antiangiogenic strategies in GEA.

Study name	Phase	Disease Setting	Investigational arm	Planned accrual	ClinicalTrials.gov Identifier	Status
ARMANI*	III	First-line, HER-2 negative GEA	Paclitaxel + Ramucirumab	280 patients	NCT02934464	Recruiting
REGONIVO*	III	Third-line, refractory GEA	Regorafenib^1^ + Nivolumab^2^	450 patients	NCT04879368	Recruiting
INTEGRATE II*	III	Third-line, refractory GEA	Regorafenib^3^	250 patients	NCT02773524	Active, not recruiting
REGOMUNE*	I/II	Solid tumors (cohort C: GEA)	Regorafenib^4^ + Avelumab^5^	482 participants	NCT03475953	Recruiting

^1^Orally at a dose of 90mg (3x30mg) qd, d1-21, q4 weeks 28-day.

^2^Intravenously at a dose of 240 mg d1 q2 weeks; after 2 months, patients whose disease is controlled may have nivolumab administered 480 mg q4 weeks.

^3^Orally at a dose of 160 mg (4x40mg) qd, d1-21, q4 weeks.

^4^Orally at a dose of 160 mg (4x40mg) qd, d1-21, q4 weeks.

^5^Intravenously at a dose of 10 mg/kg every 2 weeks, starting at Cycle 1 Day 15.

*Database accessed on 29^th^ July.

## The challenge of biomarkers discovery

In light of the conflicting results achieved so far by the targeting of angiogenesis in GEA, the search for biomarkers predictive of response/resistance has long been pursued to identify patients more likely to benefit from this approach.

Until now, only a few angiogenic factors have been linked to the prognosis of GEA, among which the potential role of VEGF isoforms has been analyzed in many studies. As such, Wang et al. reported that preoperatively VEGF-C levels in serum are significantly higher in patients who develop lymph nodes and distant metastases (81). Then, in a study by Tsirlis et al. (82) it has been shown that in resectable gastric cancer, presurgical VEGF-C levels were lower and VEGF-D levels were higher compared with healthy controls. After surgical resection, an increase in VEGF-C levels and a simultaneous decrease in VEGF-D levels was seen as compared to the preoperative scenario. Notably, both serum VEGF-C and VEGF-D levels were independent predictors of the presence of gastric cancer, with an optimal predictive model of 88% sensitivity and 83% specificity. Therefore, the VEGF-C/VEGF-D ratio was identified as the most robust predictor of malignancy with 88% sensitivity and 75% specificity, by using a cut-off value of <2.7. The authors also identified a predictive model that included VEGF-D, Ca19-9, and demographic parameters (sex, age) for the presence of lymph node metastases with 85% accuracy. Similar results were reported in another study (83), where patients displaying the expression of VEGF-C had a poorer mean survival than those without the expression of the marker (33.8 ± 13.3 vs 42.6 ± 7.4 months, p<0.001). Taken together, these findings suggest that a high expression of VEGF-C in the serum of healthy individuals is a promising diagnostic marker for predicting GEA risk, while in patients with a known diagnosis of GEA, VEGF-C levels could predict an unfavourable prognosis after surgery.

In a further study, Kikuchi et al. (84) showed that VEGF serum levels were significantly higher in patients with cancer than in healthy controls, while the levels of VEGFR-1 and VEGFR-2 were lower in the former versus latter group. VEGF was particularly sensitive and specific as a marker associated with intestinal-type cancer. Indeed, VEGFR-1 had the highest sensitivity and specificity for early gastric cancer and VEGFR-2 for diffuse-type and advanced cancer. According to the authors, a possible reason of the reduced VEGFR serum levels was that the antibody used for ELISA analysis could recognize the same or a near region as the ligand binds. High VEGF levels may bind to these receptors, thus reducing the VEGFR-1 and -2 serum levels. As vascularization promotes cancer progression, soluble VEGFR-1 and -2 could act as decoys impeding VEGF-VEGFR-2 binding on the surface of target cells.

Of interest, the circulating levels of the isoform VEGF-A were significantly higher in both serum and plasma from GEA patients. This value has been shown to decline upon curative-intent surgery, suggesting its secretion by the tumor mass (85). This study highlights a potential role of this biomarker in assessing the completeness of surgical excision.

In another study, Park and colleagues looked at ethnical differences regarding GEA characteristics and VEGF-A expression between Caucasian and Asian patients (86). They were able to find that serum levels of VEGF-A were twice as high in the former than in the latter patients’ group and that VEGF-A was an independent prognostic factor for OS, though only among non-Asian patients: the 5-year OS for VEGF-A-high vs –low patients was 72 vs 43% among Caucasians (p=0.001) and 86 vs 77% among Asians (p=0.236).

In the advanced disease setting, a correlation between serum VEGF and platelet counts has been reported, together with worse outcome in patients expressing high serum VEGF per platelet count (87). In the study of Seo et al., the OS was poorer in patients harboring high VEGF per platelet count (p=0.0432), as was the mPFS (4.5 vs. 8.9 months; P = 0.0116). This comes with no surprise, as peripheral blood cells, such as platelets, granulocytes, and lymphocytes normally express VEGF and this is released during clotting upon platelet activation. However, in the multivariate analysis for predictors of OS and PFS, a statistically significant role for VEGF per platelet was confirmed only on PFS.

Other experiences have been reported on the potential biomarker role of Ang 1-4 and Endostatin, which act as negative regulators of angiogenesis. In a review of Wang et al. (88), an increased level of endostatin in patients with gastric cancer, especially in those with lymph node invasion, as compared to healthy controls, and a lower serum level in low-grade tumors was found. These results suggest the link between serum Endostatin levels and the biological aggressiveness of cancer. Endostatin has also been shown to contribute to lymph node dissemination and to represent a prognostic biomarker in patients with metastatic GEA. Other studies showed a possible correlation between Endostatin serum levels and the histologic intestinal type of gastric tumors (89) and a significant correlation with the presence of distant metastases (90).

On the other hand, Engin et al. (91) found that the concentrations of Ang-2 and Tie-2 were significantly more elevated in patients with gastric cancer as compared to controls, even if this difference was not statistically significant across cancer stages. Jo et al. (92) confirmed this result, also showing that Ang levels were strongly associated with the presence of lymph node metastasis. Similarly, the Ang-like family protein has been explored. The Ang-like 2 (ANGPTL2), which regulates angiogenesis, has been found at higher levels in patients with gastric cancer than in controls (93). Its expression has also been correlated with tumor progression, early recurrence, and poor prognosis (94).

In the study of Oh et al. (95), both p53 and HIF-1α proteins were positively linked to depth of invasion, and p53 and VEGF levels were associated with lymph node involvement. HIF-1α was also identified as a negative prognostic factor for disease recurrence and progression.

Finally, the well-known role of some Interleukins (IL) as pro-angiogenic chemokines has led to the identification of IL-8 as a potential biomarker of angiogenesis in gastric cancer by inducing the overexpression of the VEGF-A, and the receptor isoforms VEGFR-1, and VEGFR-2 (96).

However, to date, these biomarkers have been not been validated for use in clinical practice. Furthermore, there are no predictive biomarkers also for antiangiogenic agents, even if some retrospective studies provided interesting data. In the REGARD exploratory biomarkers analysis, the authors evaluated patients’ serum and tumor samples, aiming at correlating ramucirumab efficacy with biomarker expression, including VEGFR family, HER2, VEGF-C and -D. Even if high VEGFR-2 endothelial levels were correlated to a shorter PFS (high vs low HR = 1.65) and ramucirumab treatment was associated with an improved OS in both high (HR = 0.69) and low (HR = 0.73) VEGFR-2 subgroups, none of these and other correlations reached the level of statistical significance (97). A similar conclusion was given by Van Cutsem et al. in the biomarker analyses from the RAINBOW trial (98). However, in this analysis, some pharmacodynamic and prognostic relationships were found, such as the increase of VEGF-D and PlGF plasma levels from baseline levels during treatment and their decrease after treatment discontinuation. In contrast, Angiopoietin-2 exhibited a decrease during treatment, then increased after treatment discontinuation. In this setting, also in a retrospective Korean study (99) an exploratory analysis was conducted to identify potential biomarkers predictive of response to ramucirumab plus paclitaxel in GEA patients. The authors investigated both molecular characteristics on tumor tissue (e.g. EBV, MMR, EGFR, HER2, C-MET) and circulating biomarkers (VEGF, VEGFR-2, neuropillin-1, IL-8, and PIGF) before and after treatment in a subset of patients 44 and 14 patients, respectively. They found a longer PFS in patients with higher pretreatment serum levels of VEGFR-2 (4.1 vs. 2.4 months, p=0.01) and lower pretreatment serum levels of neuropillin-1 (5.8 vs. 2.4 months, p< 0.01), suggesting that circulating angiogenesis-related biomarkers may predict prolonged response to Ramucirumab. Finally, Natsume et al. identified PIGF as a significant gene with an aberrant expression between Ramucirumab responders and non-responders (100). In fact, both the OS and PFS were significantly shorter in the PlGF-high compared with the PlGF-low group, with an ORR of 50% in the latter versus 0% in the former. Furthermore, the authors showed that genetic silencing of PIGF significantly enhanced the inhibitory effect of ramucirumab cell lines of gastric cancer co-cultured with endothelial cells. Moreover, Hacker et al. evaluated the role of Ang-2 as a prognostic and/orpredictive biomarker of bevacizumab efficacy from the AVAGAST trial (101). The median baseline plasma Ang-2 level was lower in Asian vs non-Asian patients (P<0.0001), and was identified as an independent prognostic marker for OS and correlated with the frequency of liver metastasis. However, it did not predict bevacizumab efficacy neither alone nor in combined with baseline VEGF.

## Novel developments and future perspectives

As reported before, considering the great efficacy of the combinations of anti-angiogenic drugs with immunotherapy in different cancer entities like non-small-cell lung cancer, hepatocarcinoma, and renal cell cancer, this approach is being evaluating also in gastric cancer. In the REGONIVO study, the combination of regorafenib and nivolumab showed great activity (ORR = 44%) in patients with gastric cancer progressed to prior therapy. Furthermore, a phase I trial showed encouraging ORR and DCR in patients treated with ramucirumab plus pembrolizumab (102).

In the EPOC1706 trial, patients with advanced GEA received lenvatinib in combination with pembrolizumab as first- or second-line therapy. Objective remission was reached in 20 patients out of 29, with an ORR of 69%, mPFS of 7.1 months (95% CI: 5.4-13.7), and mOS not achieved (95% CI: 8-11 months not achieved) (103). The combination of ramucirumab with pembrolizumab was explored in a multi-cohort phase 1B trial of GEA patients; it showed an ORR of 7% and a DCR of 44% (104). In a similar cohort, the mPFS was 2.6 months and OS was 12.4 months in patients treated with ramucirumab/durvalumab in the unselected population, with enhanced activity observed in patients with high PD-L1 expression (105). Finally, another phase I/II clinical trial is evaluating the combination cabozantinib plus durvalumab in pretreated patients with advanced GEA. The authors reported an mPFS of 3.8 months (95% CI: 3.4–6.3), a mOS of 9.1 months (95% CI: 5.8–21.8), and a 6-month PFS rate of 34.5% (95% CI: 17.9–54.3) (104).

One of the most actively investigated subjects in cancer research is represented by microRNAs (miRNA). They consist of a group of small non-coding transcripts, 18-24 nucleotides in length, that regulate gene expression at the translational level, being involved in a plethora of biological functions including proliferation, apoptosis, embryonic stem cell advancement regulation, and cancer cell invasion (106, 107). In particular, a growing body of evidence has been showing that miRNAs can affect tumor angiogenesis through the targeting of both pro- and anti-angiogenic factors, including, among others, RTK signalling proteins, HIF, VEGF, and epidermal growth factor (EGF) (108). To this end, MiR-616-3p has been demonstrated to promote angiogenesis in many cancers, including gastric cancer, via the PTEN/AKT/mTOR pathway (109). In a study performed on patients who underwent upfront surgery, the authors investigated miR-616-3p expression in tumor/normal tissues using real-time PCR and found a significant miR-616-3p up-regulation in cancerous tissues compared with their normal counterparts. Furthermore, miR-616-3p was associated with poor relapse-free survival (RFS) and OS in gastric cancer patients, suggesting its role as a potential prognostic marker in this cancer. Finally, they showed that miR-616-3p promoted epithelial-to-mesenchymal transition (EMT) and angiogenesis in vitro, by modulating VEGF-A and VEGFR-2 expression, and down-regulating PTEN, which resulted in the activation of the AKT/mTOR pathway. In contrast, the reduced expression of miRNA-126 facilitates angiogenesis through its interaction with VEGF-A in gastric cancer (110). Similarly, the diagnostic, prognostic and predictive value of other miRNAs in gastric cancer has been described (111). In a comprehensive review, the authors identified five groups of miRNAs, according to their referral pathway: 1) miRNAs involved in the VEGF pathway, as the previously mentioned miR-616-3p and miR-126, but also miR-1, whose overexpression significantly decreases VEGF-A and endothelin-1 expression, miR-27b, miR-101 and miR-128, that downregulate VEGF-C expression. 2) miRNAs involved in the HIF pathway, such as miR-574-5p and miR-210, are upregulated in hypoxic conditions in gastric cancer. 3) miRNAs related to HGF/c-MET signaling, such as miR-26a/b, are downregulated both in vitro and in vivo in gastric cancer, when HGF is upregulated. 4) miRNAs involved in the PI3K pathway, with different targets, such as PTEN (miR-23°, miR-616-3p, miR-718 and miR-382), FOXO (miR-155 and miR-135b), mTOR (miR-18°, miR-17-92, and miR-101-2), and NF-kB (miR-532-5p). 5) miRNAs related to STAT-3 signaling, such as miR-874, whose upregulation inhibits STAT-3 gene expression, resulting in the inhibition of VEGF-A expression and reduction in tumor growth and angiogenesis in gastric cancer.

With the development of liquid biopsy technology and exosome research, researchers have increasingly investigated the link between exosomes and circular RNAs (circRNA) in tumors. These circRNAs in exosomes could represent biomarkers for tumor diagnosis (112). Furthermore, exosomes are essential mediators of metastasis and angiogenesis in the tumor microenvironment. Some of them are deeply associated with VEGF. In fact, Xie et al. found that exosomal circSHKBP1 can promote gastric cancer cell proliferation, invasion, migration and angiogenesis by regulating the miR-582-3p/HUR/VEGF pathway (113).

Owing to the relevant biological role played by angiogenesis-related ncRNAs, they represent a promising cancer biomarker. Furthermore, some of these miRNAs may be associated with the benefit and toxicity of antiangiogenic agents, even if a prospective validation is needed to confirm these data.

More recently, several studies concentrated efforts on optimizing antiangiogenic therapeutic strategies. Shu et al. investigated the role of branched-chain amino acid transaminase 1 (BCAT1) in the pathogenesis of gastric cancer, particularly in angiogenesis (114). BCAT1 is the predominant isoform of BCAT that initiates the catabolism of branched-chain amino acids and has been involved in tumor pathogenesis, including breast cancer (115), leukaemia (116), ovarian cancer (117), glioma (118), and nasopharyngeal carcinoma (119). BCAT1, which act as an oncogenic factor, has been implicated in proliferation, invasion, and angiogenesis via the activation of the PI3K/AKT/mTOR pathway, The authors discovered that the overexpression of BCAT1 in gastric cancer patients was associated with a poor prognosis. These findings could represent the basis for a possible therapeutic target in this cancer, optimizing antiangiogenesis strategies.

Novel approaches focus on inhibiting gastric cancer vasculogenic mimicry (VM), which plays an essential role in regulating the progression and metastasis (120). The mechanism underlying VM formation is unclear, so there is a need for targeted therapies. Two recent works have recently been published on this topic. In the first, the role of Tenascin-c (TNC) has been explored (121). It is an extracellular matrix protein that induces VM in glioma (122), melanoma (123), and gastric cancer, which is strongly expressed and associated with poor prognosis (124). Qi et al. showed that gastric cancer patients displaying higher levels of TNC experienced shorter OS and RFS compared to those with low expression. They also found that the TNC knockdown inhibited the VM formation both in vitro and in vivo and suppressed the proliferation of gastric cancer cell lines through the induction of cell cycle arrest in the G0/G1 phase. Then, they discovered a strong association between TNC and EMT, which is involved in VM formation through MAPK/ERK pathway (125, 126). For this reason, the authors stopped the EMT process by blocking ERK phosphorylation and TNC, inhibiting the VM formation simultaneously. Therefore, they pointed out that the combined targeting of TNC and ERK pathways may provide a promising antitumor strategy for inhibiting VM formation and decreasing antiangiogenic therapeutic resistance.

In a second work (127), the authors investigated the role of crocetin, a component of saffron stigma, which has significant therapeutic effects on various diseases, including cancers. They showed that crocetin significantly inhibited angiogenesis and metastasis development in gastric cancer by inhibiting Human Umbilical Vein Endothelial Cells and VM formation of cells. This ability was mediated by the inhibition of the sonic hedgehog signalling pathway, making crocetin a potentially effective therapeutic drug against this cancer.

## Author contributions

MS, FG, and FP conceived the topic and MS, FC, AB, SC, CB, FP, MD, and FG wrote this manuscript. MS, FG, MD, and FP revised the manuscript. All the authors contributed to this article and approved the final submitted version.

## Conflict of interest

The authors declare that the research was conducted in the absence of any commercial or financial relationships that could be construed as a potential conflict of interest.

The handling editor AP declared a past co-authorship with the authors.

## Publisher’s note

All claims expressed in this article are solely those of the authors and do not necessarily represent those of their affiliated organizations, or those of the publisher, the editors and the reviewers. Any product that may be evaluated in this article, or claim that may be made by its manufacturer, is not guaranteed or endorsed by the publisher.
